# Long-range magnetic coupling across a polar insulating layer

**DOI:** 10.1038/ncomms11015

**Published:** 2016-03-16

**Authors:** W. M. Lü, Surajit Saha, X. Renshaw Wang, Z. Q. Liu, K. Gopinadhan, A. Annadi, S. W. Zeng, Z. Huang, B. C. Bao, C. X. Cong, M. Venkatesan, T. Yu, J. M. D. Coey, Ariando, T. Venkatesan

**Affiliations:** 1 NUSNNI-Nanocore, National University of Singapore, Singapore 117411, Singapore; 2 Department of Physics, National University of Singapore, Singapore 117542, Singapore; 3 Division of Physics and Applied Physics, School of Physical and Mathematical Science, Nanyang Technological University, Singapore 637371, Singapore; 4 Department of Pure and Applied Physics, Trinity College, Dublin 2, Ireland; 5 Department of Electrical and Computer Engineering, National University of Singapore, Singapore 117576, Singapore

## Abstract

Magnetic interactions in solids are normally mediated by short-range exchange or weak dipole fields. Here we report a magnetic interaction that can propagate over long distances (∼10 nm) across a polar insulating oxide spacer. Evidence includes oscillations of magnetization, coercivity and field-cooled loop shift with the thickness of LaAlO_3_ in La_0.67_Sr_0.33_MnO_3_/LaAlO_3_/SrTiO_3_ heterostructures. Similar modifications of the hysteresis loop appear when two coupled films of La_0.67_Sr_0.33_MnO_3_ are separated by LaAlO_3_, or another polar insulator, but they are absent when the oxide spacer layer is nonpolar. The loop shift is attributed to strong spin–orbit coupling and Dzyaloshinskii–Moriya interaction at the interfaces. There is evidence from inelastic light scattering that the polar spacer mediates long-range transmission of orbital magnetization. This coupling mechanism is expected to apply for any conducting ferromagnetic oxide with mixed valence; in view of electron hopping frequency involved, it raises the prospect of terahertz tunability of magnetic coupling.

## Abstract (web summary)

Magnetic interactions in solids are usually short-range or else they involve itinerant electrons. Here, the authors evidence a long-range magnetic coupling mediated by orbital moments in a polar spacer layer of nonmagnetic insulating oxide, with a sign which oscillates with spacer thickness.

## Graphical abstract

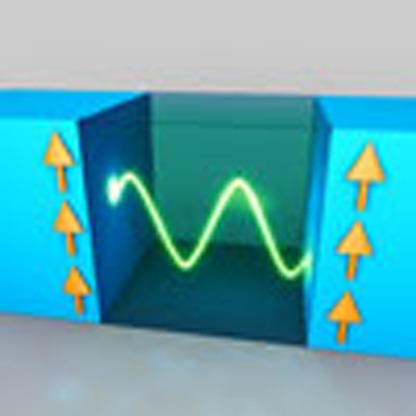

Exchange interactions in insulators usually involve virtual transfer of spin-polarized electrons in a process known as superexchange. The magnetic interaction between an atom with spin **S**_i_ at the origin and another **S**_j_ at distance *r* is described by the Heisenberg Hamiltonian =−2*J*_ij_ **S**_i_·**S**_j_, where the exchange constant *J*_ij_ are positive for ferromagnetic interactions and negative for antiferromagnetic interactions. Exchange interactions in oxides are often negative^1^. Since *J*_ij_ depends on the overlap integrals between the wave functions of neighbouring atoms, superexchange may be relatively strong (*JS*^2^≤10 meV) for nearest-neighbour cations, but the interaction falls off rapidly when *r*>0.5 nm (ref. 2).

Longer-ranged exchange is possible when the spin-polarized electrons are delocalized. Examples are metallic oxides, where ferromagnetic direct or double exchange is important^3^, and thin-film structures such as spin valves, where two ferromagnetic metallic layers are separated by a metallic spacer and the exchange interaction between them oscillates in sign with spacer thickness^4^. For oxide-based systems, oscillatory exchange coupling as a function of the thickness of the spacer has been found, for example, in superlattices involving Fe_3_O_4_ and TiN (ref. 5). The TiN spacer layers are conducting, and the coupling is attributed to oscillatory spin polarization of the magnetite conduction electrons as a function of TiN layer thickness. The theory of exchange coupling in thin-film sandwich structures with conducting or insulating spacers has been developed by Bruno^6^.

Here we are concerned with the coupling of films of ferromagnetic conducting oxides across relatively thick layers of insulating oxide, where exchange is not anticipated. The spacers are (001) polar films of LaAlO_3_ (LAO) or NdGaO_3_ (NGO) where the formal layer charges are ±*e*, with *e* the elementary charge, and (001) SrTiO_3_ (STO), which is formally nonpolar since the structure is a stack of uncharged layers of SrO and TiO_2_. The ferromagnetic mixed-valence manganite La_0.67_Sr_0.33_MnO_3_ (LSMO) is also polar with a formal layer charge of ±0.67*e*, although the charge may be screened by the Mn conduction electrons as the Thomas–Fermi screening length, *l*_TF_=0.17 nm for carrier density *n*_e_=1 × 10^27^ electrons per m^3^, is comparable to half a unit-cell layer thickness, 0.19 nm.

## Results

### The configuration and growth of the heterostructures

Our heterostructures usually involve a thickness parameter *t*_f_=10 nm film of LSMO on top of a polar or nonpolar oxide spacer. On the lower side, there may be a similar LSMO layer, or else a quasi two-dimensional electron gas (2DEG) induced in an STO substrate when the spacer is LAO^7^. The upper LSMO can be replaced by a different ferromagnetic oxide, SrRuO_3_ (SRO) or La_0.67_Sr_0.33_CoO_3_ (LSCO). It is convenient to express the layer thickness *t* in these heterostructures as a multiple *n* of the cubic perovskite unit cell (u.c.) parameter *a*_0_=0.39 nm. The heterostructures grow epitaxially, with little interdiffusion of the constituents (Supplementary Figs 1 and 2)

The 2DEG that forms at the LAO/STO interface has been shown to exhibit magnetic order^8^,^9^,^10^,^11^. The coexistence of ferromagnetic, and paramagnetic or diamagnetic phases below 100 K is associated with nanoscale electronic phase separation^8^. Ferromagnetism persists up to room temperature in samples grown at high oxygen pressure. Sufficient conditions for electronic surface reconstruction and emergence of the 2DEG are that the STO should be TiO_2_ terminated and that the LAO should be >4-u.c. thick^12^.

### Magnetic coupling between LSMO and 2DEG at LAO/STO

**Figure 1:**
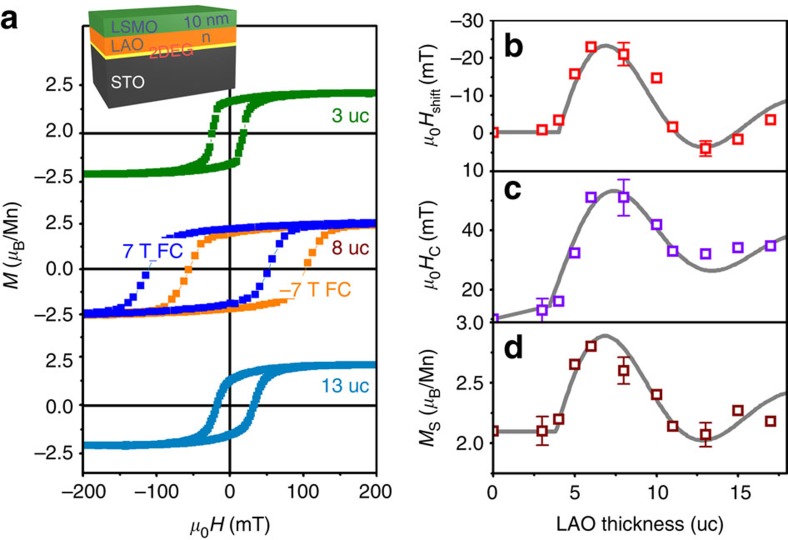
Oscillatory behaviour in La_0.67_Sr_0.33_MnO_3_/LaAlO_3_/SrTiO_3_ heterostructures. (**a**) At 10 K, hysteresis loops with different LaAlO_3_ thickness after 7 T field cooling; a reverse case is shown in second panel. (**b**–**d**) The horizontal shift of the *M–H* loop (*H*_shift_), coercivity (*H*_c_) and saturation magnetization (*M*_s_) as a function of insulator thickness, measured as a multiple *n* of the unit cell (uc) parameter (0.39 nm). The solid lines in **b**–**d** are all fits to a exponentially damped sinusoidal oscillation with period *n*−9 u.c. and attenuation length *n*=9 u.c.

Figure 1 shows the 10 K *M*–*H* hysteresis loops of 10 nm LSMO on LAO/STO, including the effect of field cooling in an in-plane field of 7 T. All the magnetic parameters (spontaneous magnetization *M*_s_, coercivity *H*_c_ and the horizontal field-cooled shift of the hysteresis loop *H*_shift_) depend on the thickness of the LAO spacer layer, provided it exceeds the threshold *n*=4. There are no changes when *n*≤4. This demonstrates that the charge transfer to the underlying STO that creates the 2DEG also modifies the magnetism of the LSMO. The magnetization of a 10-nm layer of LSMO prepared without LAO, *n*=0, is *M*_s_=350 kA m^−1^ or 2.2 *μ*_B_/Mn, where *μ*_B_ is Bohr magneton, compared with the bulk value of 3.6 *μ*_B_/Mn. The reduction of the magnetization in thin (100) LSMO films^13^ is due to a noncollinear ferromagnetic structure. The loop shift when *n*≥4 is reminiscent of exchange bias at a ferromagnetic/antiferromagnetic interface^14^, although it can be of either sign, depending on the spacer thickness. When the 7 T cooling field is reversed, so is the *M*–*H* loop shift (Fig. 1a). The maximum value *μ*_0_*H*_shift_=28 mT corresponds to an asymmetric coupling energy *σ*_a_=*μ*_0_*H*_shift_*M*_s_*t*_f_ of 0.11 mJ m^−2^. Moreover, the sign of the shift changes from negative to positive when *n*=11, and back again when *n*=16. The oscillations in magnetization, with a maximum at *n*=7 and minimum at *n*=12 are reminiscent of the oscillations in exchange found in spin valve structures as a function of the spacer layer thickness^4^, except that the period of the oscillatory behaviour in our insulating system is three to eight times greater than that found for Co/Cr/Co spin valves or magnetic tunnel junctions with an MgO barrier thinner than 3 nm (refs 15, 16). None of the explanations proposed for metallic spacers such as Ruderman–Kittel–Kasuya–Yosida or other exchange coupling via interlayer conduction electrons^17^, Fermi velocity mismatch^18^, hybridization of the interlayer conduction electrons with the interface *d*-states of the magnetic films^19^ applies to an insulating spacer. Nor can tunnelling or dipole interactions associated with correlated surface roughness (Supplementary Fig. 3) account for the magnitude of the effect^20^, or its change of sign. We propose a new magnetic interaction mechanism.

### Transport properties of 2DEG under the magnetic coupling

**Figure 2:**
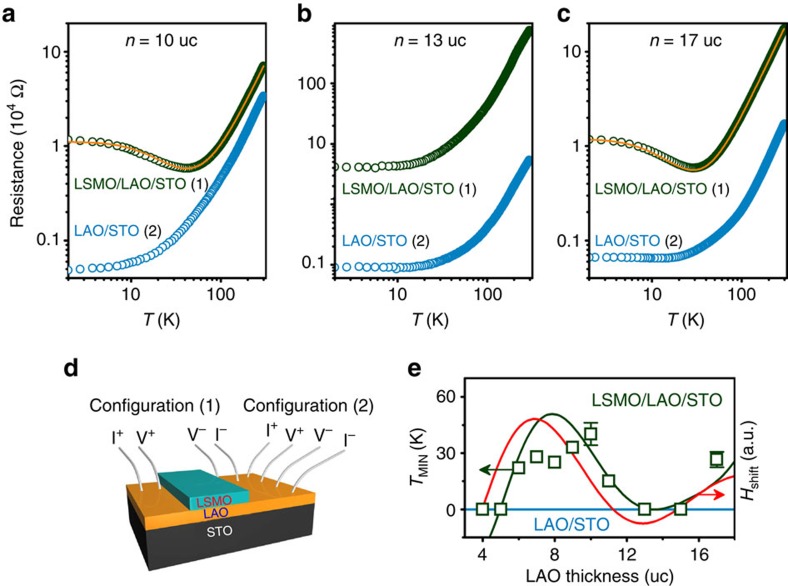
Transport properties of the 2DEG at the LaAlO_3_/SrTiO_3_ with La_0.67_Sr_0.33_MnO_3_ strip. (**a**–**c**) The resistance versus temperature for 10, 13 and 17 uc LaAlO_3_ spacers, measured in the two configurations illustrated in **d**. (**e**) The temperature of resistance minimum measured in configuration (1) as a function of LaAlO_3_ thickness.

The reciprocal interaction of LSMO with the 2DEG across the LAO spacer is demonstrated using a 3-μm strip of LSMO laid across the 5 × 5 mm^2^ (001) STO substrate. The conduction of the 2DEG was measured either parallel or transverse to the LSMO strip with four in-line contacts (Fig. 2). The insulating character of the LAO spacer was confirmed by direct electrical measurement. In the parallel case, the usual temperature dependence of conductance was found but, in the transverse geometry, a resistance minimum appears due to a marked increase of scattering at low temperature. Such minima are associated with magnetic (Kondo) scattering^21^, and are accompanied by negative magnetoresistance (Supplementary Note 1; Supplementary Fig. 4). There is no perceptible diffusion of Mn across the LAO barriers (Supplementary Fig. 2), so we are seeing an effect of the distant LSMO layer on the 2DEG, mediated by the LAO. It is the counterpart of the effect of the 2DEG on the LSMO hysteresis loop, shown in Fig. 1. The mutual interaction of the 2DEG and the LSMO is confirmed by gating experiments^22^,^23^ which show striking changes in loop shift, coercivity and resistance minimum with bias voltage (Supplementary Note 2; Supplementary Fig. 5). The changes of both magnetization and transport properties on varying the LAO thickness or gate potential indicate correlated charge transfer on opposite sides of the LAO. Electron transfer to the 2DEG, which improves its conductivity and increases the magnetic scattering, is coupled with hole depletion in the LSMO^24^,^25^, which increases its ferromagnetic moment. The magnitude of the moments on either side are coupled, but the relative orientations cannot be inferred because the 2DEG moment is only ∼1% of that of the LSMO, and too small to measure directly.

An estimate of the symmetric coupling energy between the ferromagnet and the 2DEG can be derived from the maximum change of LSMO coercivity *μ*_0_Δ*H*_c_ when the 2DEG forms. It is ∼40 mT. The change in anisotropy field *μ*_0_Δ*H*_a_ will be roughly an order of magnitude greater. The corresponding change of anisotropy energy Δ*K*_1_=*μ*_0_*M*_s_Δ*H*_a_/2 expressed per unit area is *σ*_s_≈0.8 mJ m^−2^ or 9 K*a*_0_^−2^. Since 0.5 electrons per unit cell area at the STO interface are needed to avert the polarization catastrophe, and <0.5 are ferromagnetic^26^, the energy per electron is >18 K. The additional scattering in the transverse strip geometry of Fig. 2 sets in above this temperature. A plot of the temperature of the resistivity minimum as a function of LAO thickness reveals an oscillatory variation quite similar to that of *H*_shift_ or *M*_s_ (Fig. 2e). The curves do not overlap at zero bias, but their similarity reinforces the idea that the LAO is transmitting magnetic information from the LSMO to the 2DEG.

### The effect of polar or nonpolar insulator

**Figure 3:**
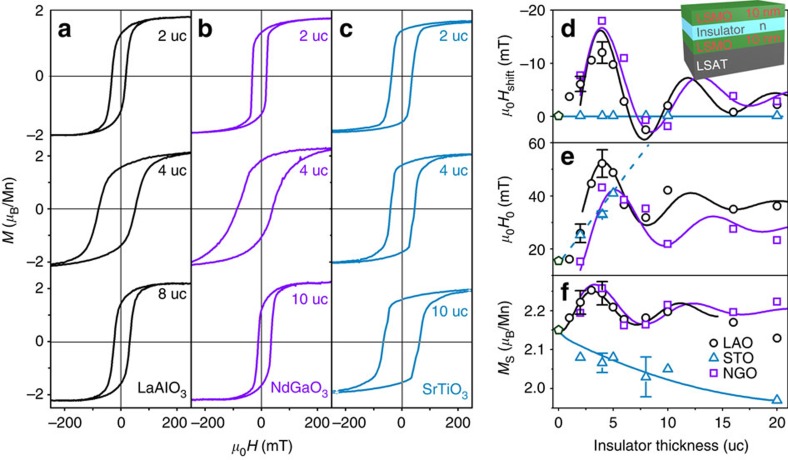
Heterostructures with polar and nonpolar insulators. (**a**) LaAlO_3_. (**b**) NdGaO_3_ and (**c**) SrTiO_3_. (**d**–**f)** The variation of horizontal shift of the *M–H* loop (*H*_shift_), coercivity (*H*_c_) and saturation magnetization (*M*_s_) as a function of insulator thickness. The data are measured at 10 K.

To investigate the magnetization further, sandwich structures consisting of two 10 nm films of LSMO separated by a layer of LAO, NGO or STO of varying thicknesses were prepared to compare the effects of polar and nonpolar barriers. Results shown in Fig. 3 for a polar insulator, LAO or NGO, resemble those already described for the LSMO/LAO/STO heterostructures, except there is now no threshold thickness. The two 10 nm films of LSMO switch together, although thinner films show evidence of independent switching (Supplementary Note 3; Supplementary Fig. 6), which demonstrates that they are not identical. The *M*–*H* loop again varies with barrier thickness in an oscillatory manner. The behaviour with a nonpolar STO spacer is quite different. Then, there are no oscillations and no loop shift, only a monotonic increase of coercivity and decrease of magnetization with *n*. To mediate oscillatory coupling, it seems that the spacer must be polar. The essential differences between polar and nonpolar oxide layers are the possibilities of charge transfer with electronic interface reconstruction, and oscillations or standing waves involving the sheets of positive and negative charge, that distinguish the (001) direction normal to the sheets in a thin film from the in-plane directions.

## Discussion

We associate the loop shift with the interface charge that is responsible for the variation of the moment with spacer thickness^24^,^25^. The symmetry-breaking interaction is likely to be the Dzyaloshinskii–Moriya (D–M) interaction **D**_ij_·(**S**_i_ × **S**_i_), where **D**_ij_ is the D–M constant, associated with enhanced spin–orbit interaction at the charged interface^27^. Orbital currents associated with geometric frustration of triangles of spins at the manganite interface^28^ may be involved. The shift oscillates as a function of LAO thickness with the oscillating interfacial charge, being greatest when electron depletion at the LAO/LSMO interfaces is the greatest and the most screening charge appears in the LSMO^24^,^25^. The direction of the loop shift depends on both the sign of the interfacial charge and the direction of magnetization of the LSMO. If the sign of the charge at the two LAO interfaces is opposite, **D**_ij_ is directed in the same sense perpendicular to the interfaces. The interface charging effects are maximum at about 5 u.c. and minimum near 10 u.c., as seen in Fig. 3, but the effects are weaker than those for a single LSMO layer and the strongly polar LAO/STO interface that we discussed first. The opposite sign of the charge at the two LSMO interfaces is attributed to the different growth conditions of the upper and lower LSMO films.

The fact that we never observe a loop shift for a single 10 nm LSMO layer in the absence of interface charge, no matter how we prepare it (Supplementary Fig. 6), points to the existence of some mechanism that couples not only the magnitudes, but also the directions of the LSMO moments across the polar spacer. Two mechanisms for transmitting magnetic interactions can be envisaged. We ruled out dipolar interactions due to correlated interface roughness because they are much too weak (Supplementary Table 1; Supplementary Fig. 3). A long-range exchange interaction is implausible. Any exchange between partly filled *d*-orbitals in the two ferromagnetic layers due to virtual charge transfer involving the filled 2*p* band should be very weak and ferromagnetic, regardless of the polar nature of the oxide; coupling by spin-polarized electron tunnelling is expected to decay exponentially over ∼2–3 u.c. (refs 6, 15, 16).

An alternative idea is a coupling mechanism that is not based on spin, but depends on the transmission of orbital correlations across the polar barrier. Electron hopping in mixed-valence^3^ manganites is a pair-localized process involving the fourth, majority spin Mn *e*_g_ electron, with an associated polaronic lattice distortion^29^. The hopping frequency *f* is of order 10^13^ Hz (refs 3, 30), and an orbital moment *m*_orb_≈π*d*^2^e*f*/8 is created in LSMO as the electron hops back and forth in the exchange field, where *d* is the Mn–Mn separation, *a*_0_/√2=0.28 nm. Planar charge excitations in the LSMO or LAO can be decomposed into two counter-rotating orbital motions, one of which could resonate with the electron hopping and couple to the Mn spins in LSMO via spin–orbit interaction. The asymmetric coupling energy of 0.11 mJ m^−2^ is equivalent to ∼1 K per interface Mn. Taking *S*=1.85 for Mn in LSMO and the spin–orbit coupling constant for Mn as Λ=150 K in the interaction Λ*LS* with a, we deduce *L*=3.7 × 10^−3^, where *L* and *S* are the orbital and spin angular momentum, respectively. This value of *L* corresponds to the orbital moment *m*_orb_/*μ*_B_ induced by electron hopping at a frequency of 7 THz.

**Figure 4:**
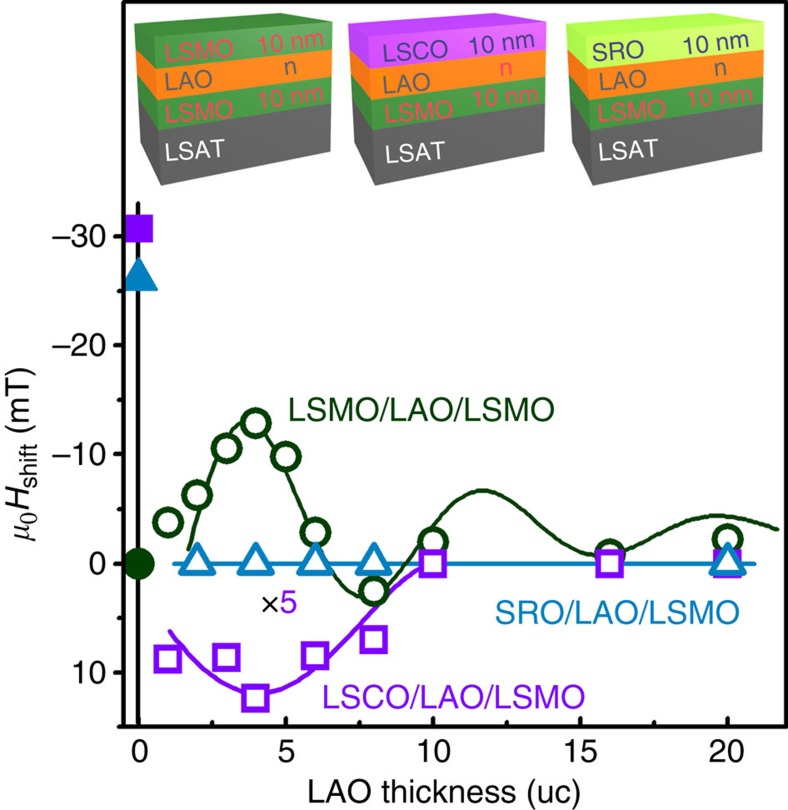
The loop shift for a 10 nm La_0.67_Sr_0.33_MnO_3_ bottom layer with different top layers. There is no shift for SrRuO_3_, which is a nonpolar metal. The shift for La_0.67_Sr_0.33_CoO_3_, which is polar but with the opposite signs of spin–orbit coupling, is opposite to that of La_0.67_Sr_0.33_MnO_3_.

Some further experiments were conducted to validate these ideas. First, the upper LSMO was replaced by SRO, which is a nonpolar ferromagnetic metal that does not exhibit pairwise electron hopping. In that case, there was no loop shift on field cooling for 5, 10 or 16 u.c. of LAO (Fig. 4), and the coercivity of the bottom LSMO layer remained essentially constant at 10–13 mT. Second, the top manganite electrode was replaced by a similar cobaltite (LSCO) electrode. The sign of the spin–orbit coupling for cobalt Λ=−272 K is opposite to that of manganese, so we would expect the sign of the loop shift to change. This is exactly what we observe (Fig. 4).

**Figure 5:**
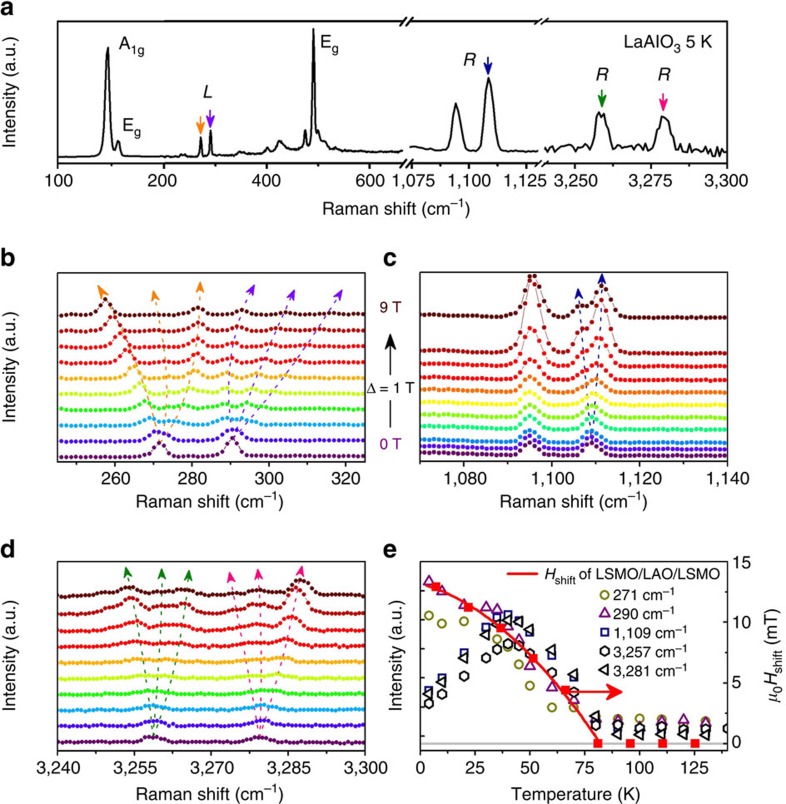
Inelastic light scattering from an LaAlO_3_ crystal at 5 K under magnetic field. (**a**) The zero magnetic field spectrum of LaAlO_3_. (**b**–**d**) The field-dependent splitting of the spectral lines up to 9 T. *R* denotes a Raman mode and *L* a luminescent transition. The transitions in **d** are third order of Raman lines in **c**. (**e**) The coincident temperature dependence of the magnetization oscillations including horizontal shift of the *M–H* loop (*H*_shift_) and the intensity of the magnetically split components in the luminescence/Raman spectrum.

To shed some light on the role of the oxide in transmitting magnetic coupling, we have studied the effect of a magnetic field on inelastic light scattering. Results shown in Fig. 5b–d are for a LAO crystal, where a number of both Raman and luminescent modes are found to exhibit Zeeman splitting. Figure 5e compares the temperature dependences of the loop shift and the intensity of the magnetic field-sensitive lines in LAO. They are similar, especially for the defect-related luminescent transitions at 272 and 290 cm^−1^, and the Raman excitations at 1,100 cm^−1^, and its harmonics which the field splits into three, corresponding to *g*=1.2±0.2. As shown in Supplementary Figs 7 and 8, the observation of similar field-sensitive excitations in other oxides correlates with the observation of a loop shift when the oxide is used as a polar spacer. The requirements for a suitable spacer oxide are that it should include a polar plane containing a heavy (rare earth) cation and oxygen, but no light cation (Sr, Al…) (Supplementary Note 4). These observations support the idea that magnetic-field-dependent excitations in a polar oxide are associated with the transmission of magnetic information across the insulating polar layer; this could involve electrons associated with Schottky defects or metal in gap states^31^ in LAO, which couple to electrons hopping at the Fermi level of LSMO. The phase of these states, possibly influenced by standing waves in the LSMO^6^ controls the oscillatory behaviour.

If the interface charge is critical, why is the oscillatory effect manifested only below 80 K, although the 2DEG is stable at room temperature? The onset temperature reflects the interlayer coupling that is mediated by orbital excitation via the LAO layer, and establishment of the phase of the gap states in the LAO. The appearance of the loop shift and magnetic oscillation only below 80 K is evidence for a role of the magnetic-field-dependent Raman and luminescent modes in the polar layer in somehow providing a coupling mechanism between the two ferromagnetic layers.

In conclusion, we have set out a range of evidence for magnetic coupling that acts across relatively thick polar layers of a nonmagnetic insulating oxide. The magnitudes of the moments are coupled by the correlated interface charge on either side of the barrier, and there is evidence that the directions of the moments are also aligned. The proposed coupling mechanism is not via interlayer exchange or dipolar interactions, but it involves orbital magnetism, which is excited at surface or defect-related optical modes in the oxide spacer. We have shown that they couple both to LSMO and to the 2DEG at the STO interface, but the modes do not couple to the nonpolar metallic ferromagnet SRO nor do they propagate in a nonpolar insulator.

## Methods

### Sample fabrication

For the LSMO/LAO/STO heterostructures, first, LAO was deposited on a TiO_2_-terminated STO substrate under 5 × 10^−2^ torr at 780 °C using pulsed laser deposition. The thickness of LAO was monitored by *in situ* reflection high-energy electron diffraction, which revealed layer-by-layer growth. Then, 10 nm of LSMO was grown under the same oxygen pressure at 720 °C. For the LSMO/oxide barrier/LSMO sandwich heterostructures, (La_0.3_Sr_0.7_)(Al_0.65_Ta_0.35_)O_3_ (LSAT) substrates were first annealed at 1,050 °C in air for 2.5 h to obtain atomically flat surfaces. Then, the LSMO (10 nm)/oxide barrier/LSMO (10 nm) heterostructures with different barrier thickness were prepared under 5 × 10^−2^ torr oxygen pressure at 720 °C. The same growth condition was also applied for SrRuO_3_ and La_0.67_Sr_0.33_CoO_3_ layer deposition. LSAT was used as the substrate because it is lattice matched with LSMO, but similar results were obtained with LAO, STO and NGO substrates (Supplementary Note 5; Supplementary Fig. 9). Exact same fabrication condition was applied in the reproducibility test (Supplementary Note 6; Supplementary Fig. 10).

### Interface structure and elementary diffusion

The interface structure was studied by high-resolution transmission electron microscopy, and a lattice image for a 10 u.c. spacer is shown in Supplementary Fig. 1. Energy-dispersive X-ray spectroscopy was scanned for elemental identification of Mn, La and Sr.

### Magnetic and transport measurement

Magnetization measurements were made using a Quantum Design superconducting quantum interference device magnetometer. Transport measurements were carried out by the Quantum Design Physical Properties Measurement System. The MR anisotropy was measured in a linear geometry as well with two different directions of the applied magnetic field, out of plane and in plane, but perpendicular to the current in each case.

## Additional information

**How to cite this article:** Lü, W. M. *et al*. Long-range magnetic coupling across a polar insulating layer. *Nat. Commun.* 7:11015 doi: 10.1038/ncomms11015 (2016).

## Supplementary Material

Supplementary Information Supplementary Figures 1-10, Supplementary Table 1, Supplementary Notes 1-6 and Supplementary References
