# Supplementary material for: Long-range magnetic coupling across a polar insulating layer
Source: Nat Commun. 2016 Mar 16;7:11015. doi: 10.1038/ncomms11015 (PMC4799368; doi:10.1038/ncomms11015)
Supplement: Supplementary Information — Supplementary Figures 1-10, Supplementary Table 1, Supplementary Notes 1-6 and Supplementary References [file ncomms11015-s1.pdf]

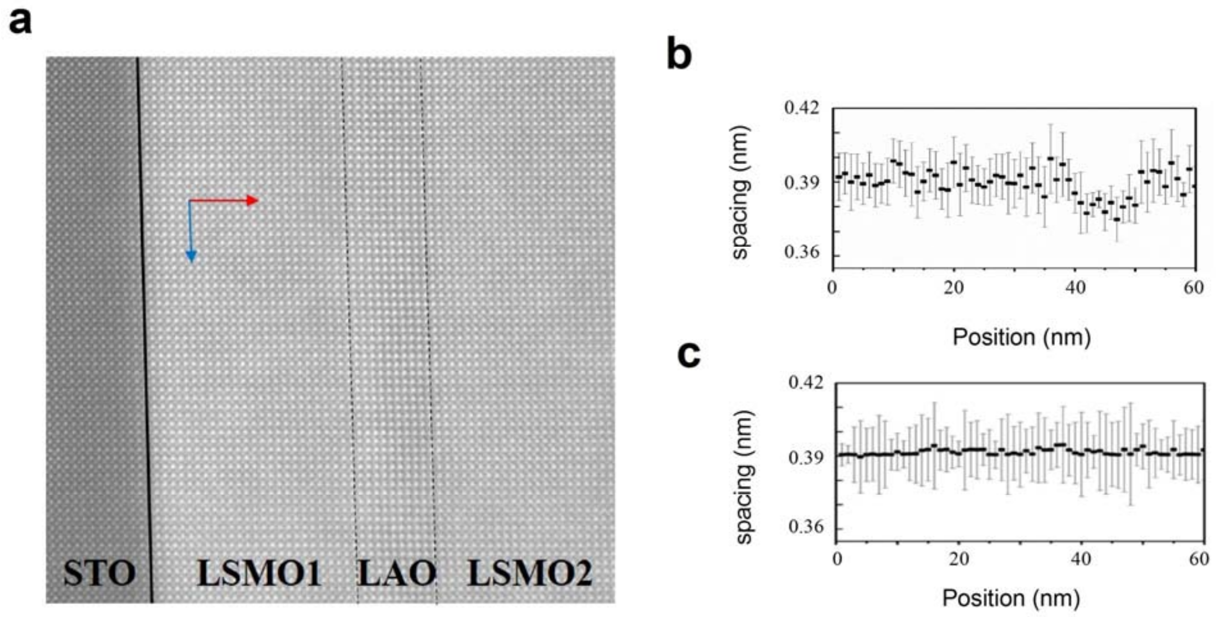

Supplementary Figure 1: The TEM cross section for LSMO/LAO/LSMO on STO with a 10 uc LAO spacer are shown in a. The line profiles, in-plane and out-of-plane marked by blue and red, are exhibited in b and c. The in-plane lattice parameter is the same for LAO and LSMO, b and c illustrates the effect of strain. For an STO substrate; the LAO is under biaxial tension with the same in-plane lattice parameter as LSMO, but there is a reduced parameter perpendicular to the plane.

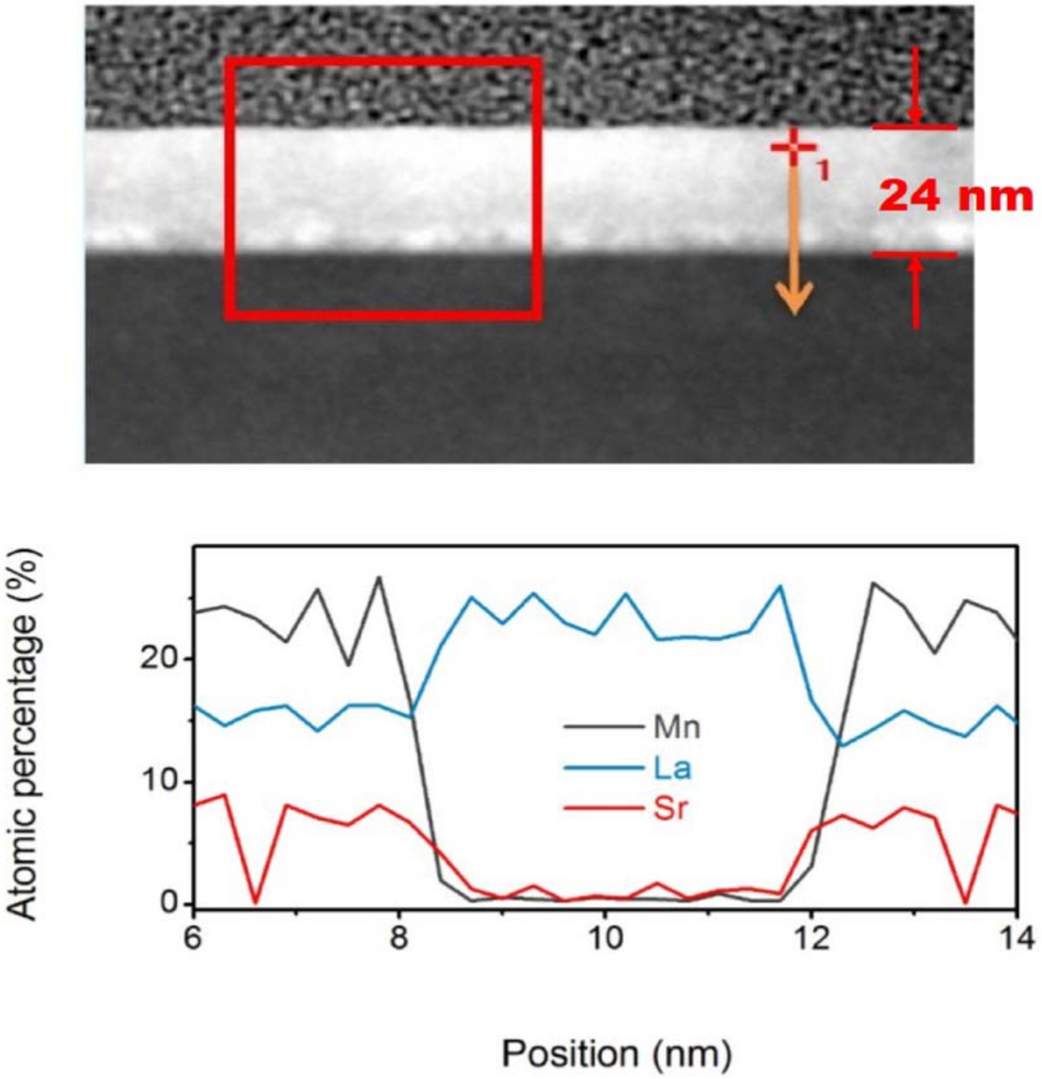

Supplementary Figure 2: TEM-EDX line profile of LSMO/LAO(10 uc)/LSMO heterostructure. A cross-sectional scan of the Mn, La and Sr profiles was obtained, it can be seen that Mn diffusion is limited to only one-two unit cells on either side of the LAO. Hence the influence of Mn on the properties of the spacer is neglected.

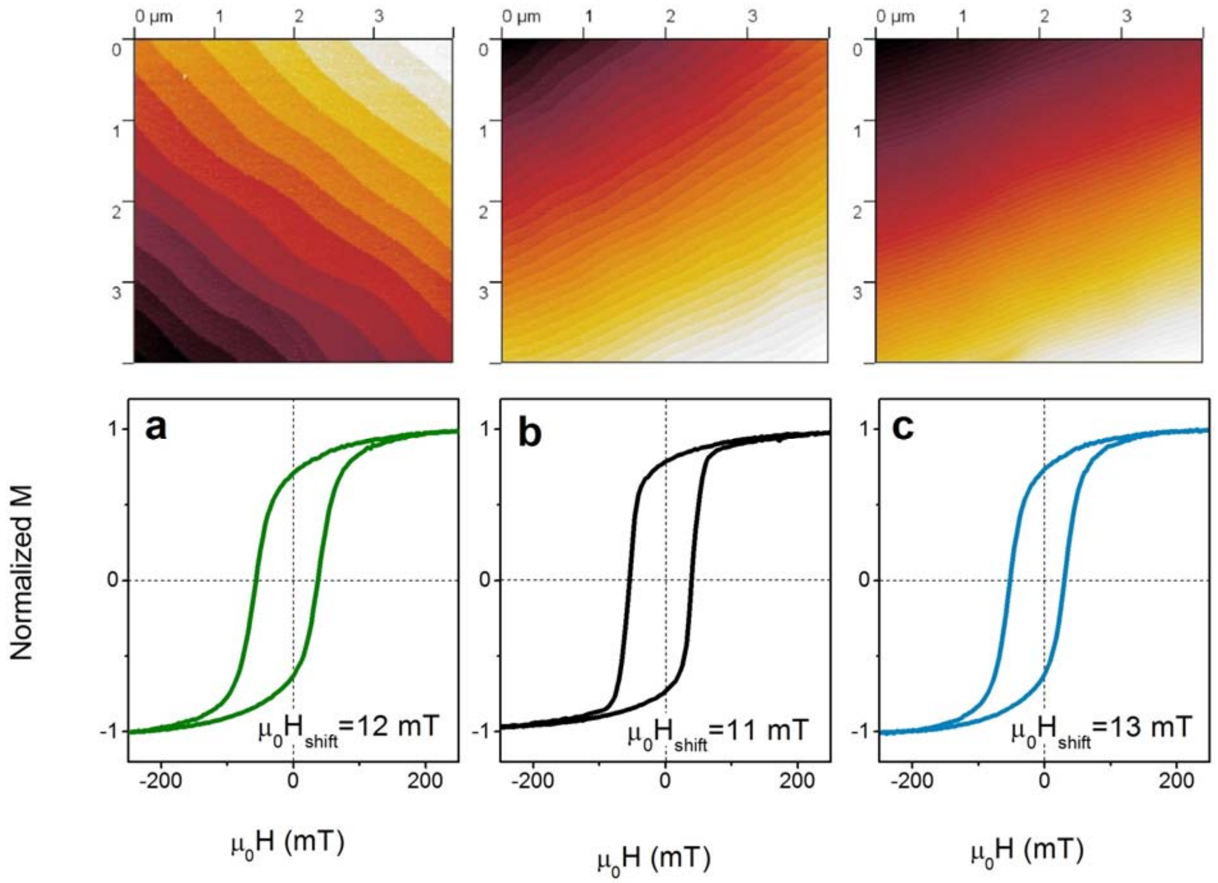

Supplementary Figure 3: The M-H curves of LSMO/LAO(4 uc)/LSMO on STO substrate with different miscut angles. LSMO sandwich on three STO substrates with different vicinal cuts is studied, where the terrace widths vary from ~70 to ~500 nm. The shift of the magnetization curves, a to c, is almost the same in all cases even though the step widths on the surface have changed from ~70 to ~500 nm. The loop shifts are similar in all three cases, 22, 18 and 24 mT, with no systematic variation. Assuming unit cell steps, a spacer thickness of 5 nm and the terrace width of 200 nm, the Kools formula [1] gives a value of σ = 0.0008 mJm^{-2}, two orders of magnitude less than the 0.11 mJ m^{-2} observed. At a spacing of 2 nm, the value is 0.0009 mJ m^{-2}. Moreover the sign does not change with spacing when $t << l$. The interaction with the 2DEG will be much weaker.

Supplementary Note 1

To characterize the low-temperature transport of LSMO/LAO/STO, magnetoresistance measurements were performed, as shown in Supplementary Figure 4. The MR anisotropy was measured in a linear geometry with two different directions of the applied magnetic field, out-of-plane and in-plane, but perpendicular to the current in each case. For the in-plane configuration, a negative MR of ~11% for LAO/STO and ~15% for LSMO/LAO/STO were observed in a 9 T at 2 K. The negative MR most likely arises from coherent scattering. The MR at 2 K for the out-of-plane field shows more interesting behavior. The LAO/STO magnetoresistance is positive, in agreement with several other reports [2, 3], over the whole field range from 0 to 9 T. There is a similar behavior of LSMO/LAO/STO under high magnetic field, but a negative MR is observed in low fields. These data establish that there is additional magnetic scattering in the 2DEG, induced by the LSMO.

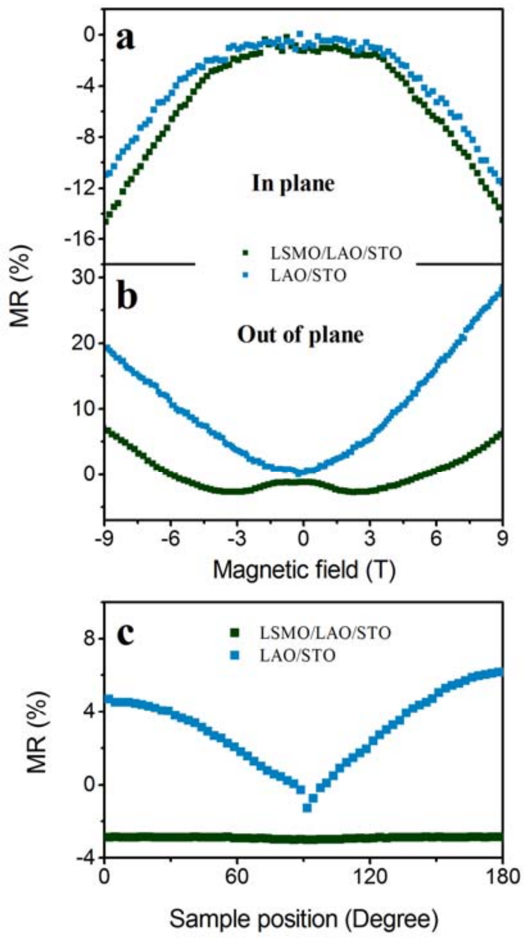

Supplementary Figure 4: Comparison of 2 K magnetoresistance between LSMO/LAO/STO and LAO/STO with magnetic field applied at different directions. a and b, in-plane MR and out-of-plane MR. c, Magnetoresistance as a function of sample orientation under 2 T magnetic field.

Supplementary Note 2

We measured the transport and magnetization properties of $La_{0.67}Sr_{0.33}MnO_3/LaAlO_3$(6 uc)/$SrTiO_3$ under electric field back gating. Supplementary Figure 5a shows the experimental configurations of gate bias measurements. With positive/negative back gate bias, as shown in Supplementary Figure 5b, enhanced low-temperature magnetic scattering is seen upon electron

injection and insulating behavior at all temperatures is seen upon electron depletion. It is seen in Supplementary Figure 5c that the hysteresis loop of LSMO is changed by back gating as well; the *M*-*H* loop shifts to higher fields with electron injection into the 2DEG, whereas M_s, H_c and H_{shift} decrease with electron depletion. A summary of H_{shift} as a function of back gating voltage is provided in Supplementary Figure 5d. The field-effect experiment suggests that not only is the magnetism of LAO/STO carrier density dependent (still a matter of dispute [4-6]) but also that the hysteresis of LSMO depends on the interface charge. The magnitudes of the moments are correlated due to the polar interlayer.

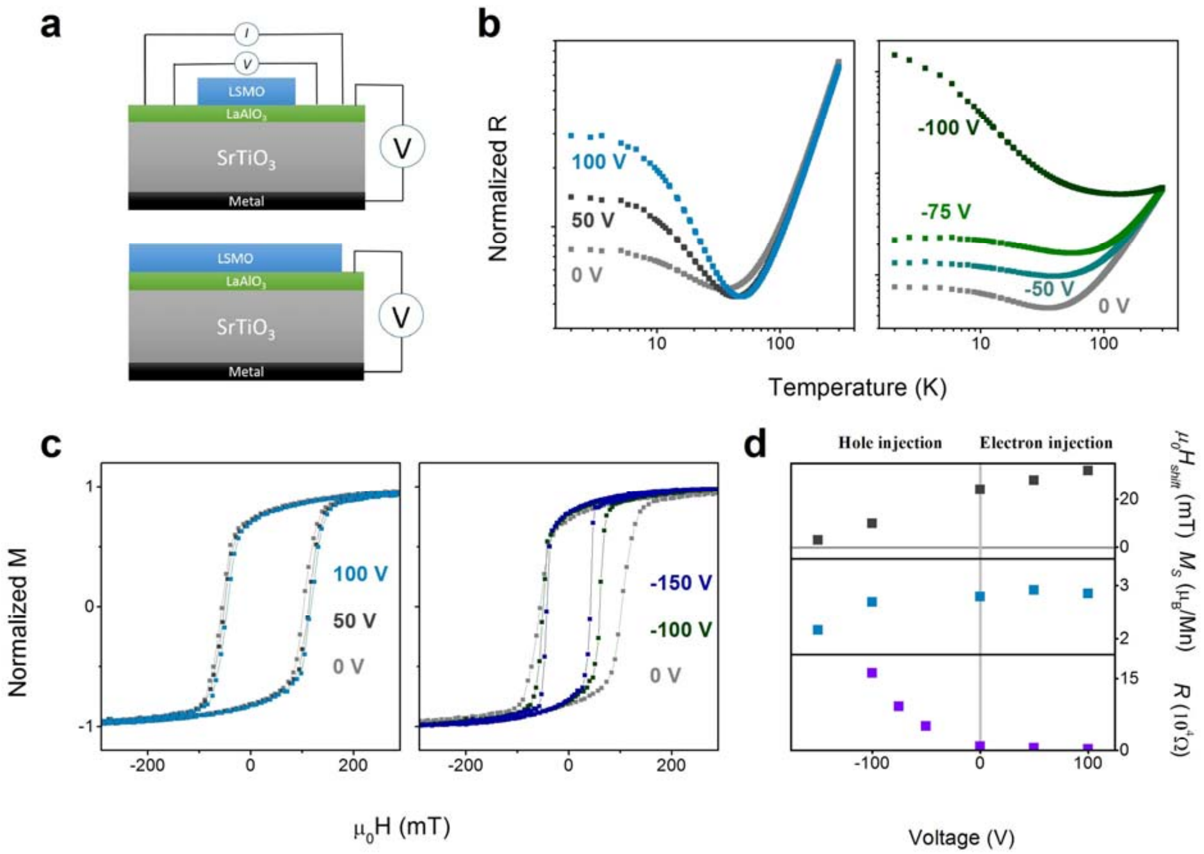

Supplementary Figure 5: Electric field back gating effect of LSMO/LAO(6 uc)/LSMO. a, The configuration for the back gate measurements. b and c, Dependence of the magnetic scattering

and the LSMO hysteresis loop on gate voltage applied across the LAO/STO heterostructures. d is a plot of H_{shift}, M_S and resistance as a function of back gate voltage.

In the gating experiments where the top electrode is wire bonded to the STO, there is electron accumulation in the 2DEG at positive bias. The 2DEG becomes increasingly conducting and magnetic, as shown by the magnetic scattering. At the same time the LAO/LSMO interface is depleted of electrons and this results in an increase in screening charge in the LSMO which favours ferromagnetic interactions and increases its magnetization. Evidence for these effects is comprehensively summarized in the reviews by Vaz. [7, 8]

Related charging effects, albeit with a phase shift, appear as a function of LAO spacer thickness. The maximum moment at n = 6 corresponds to electron depletion at the LAO/STO interface, while the maximum resistance of the 2DEG at n = 13 corresponds to an unenhanced moment of the LSMO.

Charge coupling at the two LAO interfaces leads to a coupling of the magnitudes of the moments. We suggest that this charge coupling is mediated by a metal in gap (MIG) state. [9]

Supplementary Note 3

Supplementary Figure 6 shows the hysteresis loops of single LSMO layer, capped and uncapped with LAO and sandwich heterostructures. The loops are similar, and a loop shift is never seen for a single LSMO layer, Supplementary Figure 6 d and j.

In LSMO/LAO(4 uc)/LSMO sandwich heterostructures, the thinner LSMO layers ($t \leq$ 7 nm) show different loops, which do not switch simultaneously. However when $t \geq$ 10 nm, they couple to give a single loop, and the loop shift is fully developed. Results for an 8 uc spacer show the

same trend. Although the thicknesses are the same, the top and bottom LSMO layers are actually not identical. The two layers have different outer interfaces and the inner interfaces with LAO also differ, because they have different terminations. The stack is not truly symmetric. This is evident when we take LSMO layers that are thinner than 10 nm. At 5 nm, the coercivity and magnetization of the two layers are obviously different, and they are decoupled with separate loops; the top one has larger coercivity. At 10 nm they are locked together. Furthermore, the LSMO has to be conducting for the magnetic coupling to be operative.

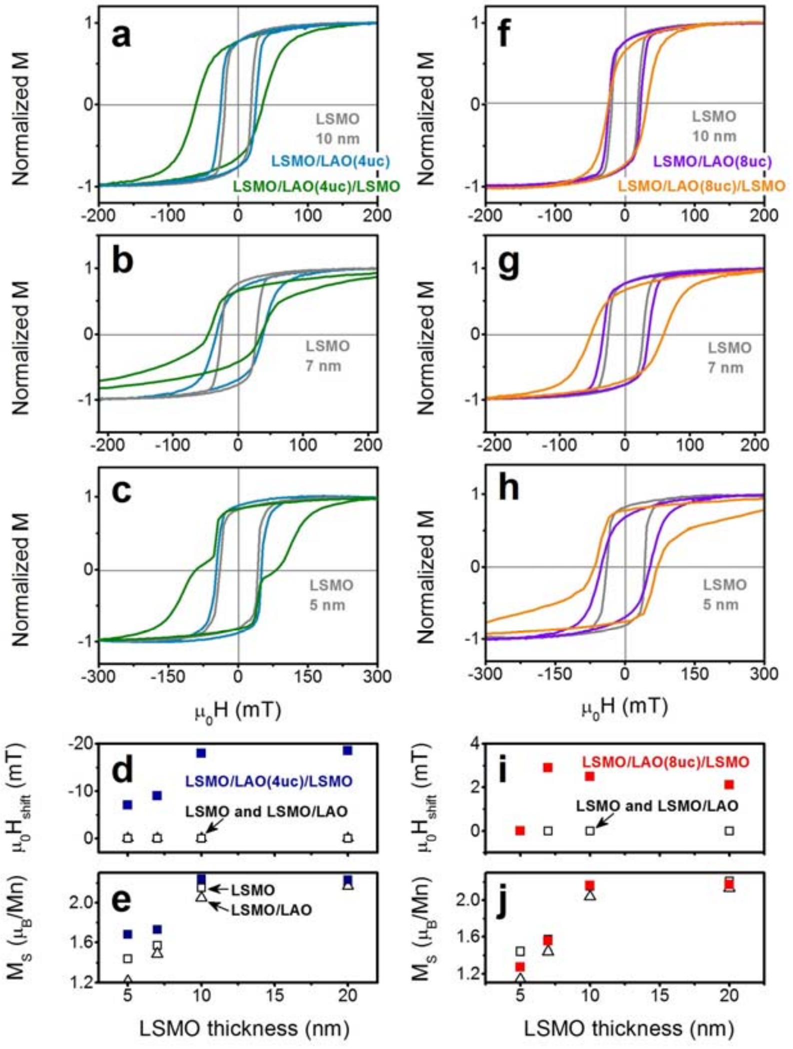

Supplementary Figure 6: Effect of changing the thickness of the LSMO layers in LSMO/LAO/LSMO heterostructures with LAO thicknesses of 4 and 8 uc. The upper and lower LSMO layers are locked together when their thickness is 10 nm.

Supplementary Note 4

Supplementary Figure 7 and 8 show the inelastic light scattering from crystals of a series of oxides which we have used as spacers in LSMO/spacer/LSMO heterostructures. Raman lines are marked with ***R***, the others are luminescent lines. The oxides which show magnetic-field-sensitive transitions all exhibit a loop shift in heterostructures. The empirical conditions for spacer oxide to mediate a long-range magnetic interaction are deduced:

1. The materials must have a polar plane.

2. In the polar plane there must be a heavy element and oxygen (e.g., LAO, NGO, DSO, NAO etc...)

3. That plane should not include any light cation (e.g., LSAT).

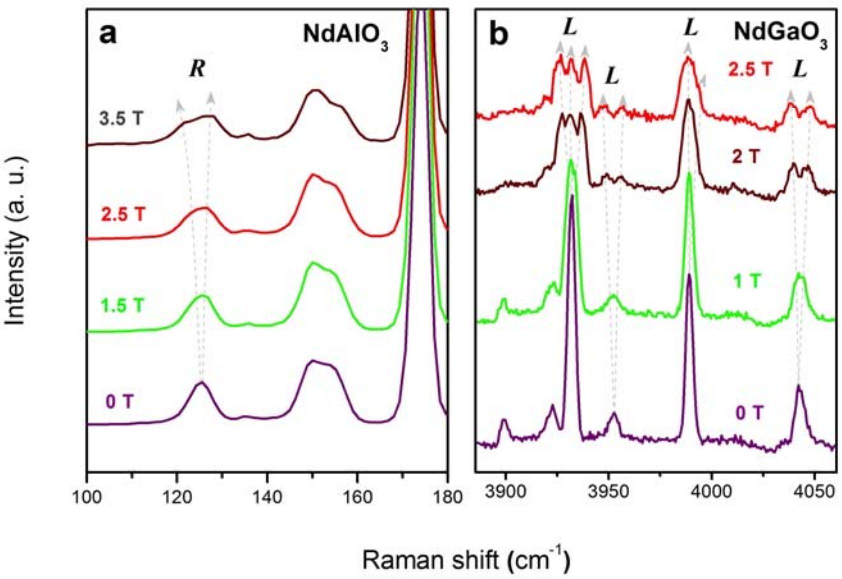

Supplementary Figure 7: The inelastic light scattering spectra as a function of magnetic field of other polar insulators at 5 K, a, $DyScO_3$ and b, $NdGaO_3$. Raman and luminescence lines are marked R and L.

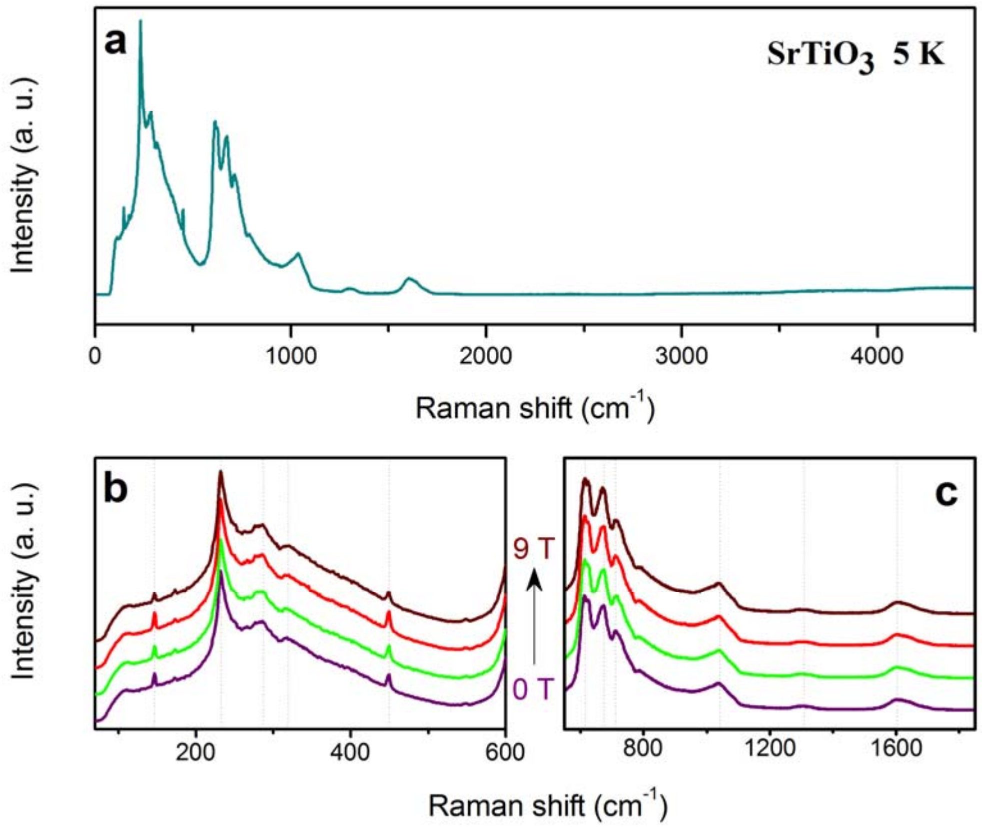

Supplementary Figure 8: The inelastic light spectrum of the non-polar insulator $SrTiO_3$. a, zero magnetic field Raman spectrum of $SrTiO_3$ at 5 K. b and c, Field dependent spectra showing absence of any magnetic field dependent features at 5 K.

Supplementary Note 5

The data in Supplementary Figure 9 show that a 10 nm LSMO/LAO/LSMO sandwich with a 4 uc LAO spacer exhibits a single shifted loop when grown on all three substrates; apparently the lattice parameter or polar character of the substrate is not critical. All sandwich stacks discussed in the text are grown on LSAT, chosen because it is lattice matched to LSMO. Of course, those involving the 2DEG have to be grown on STO.

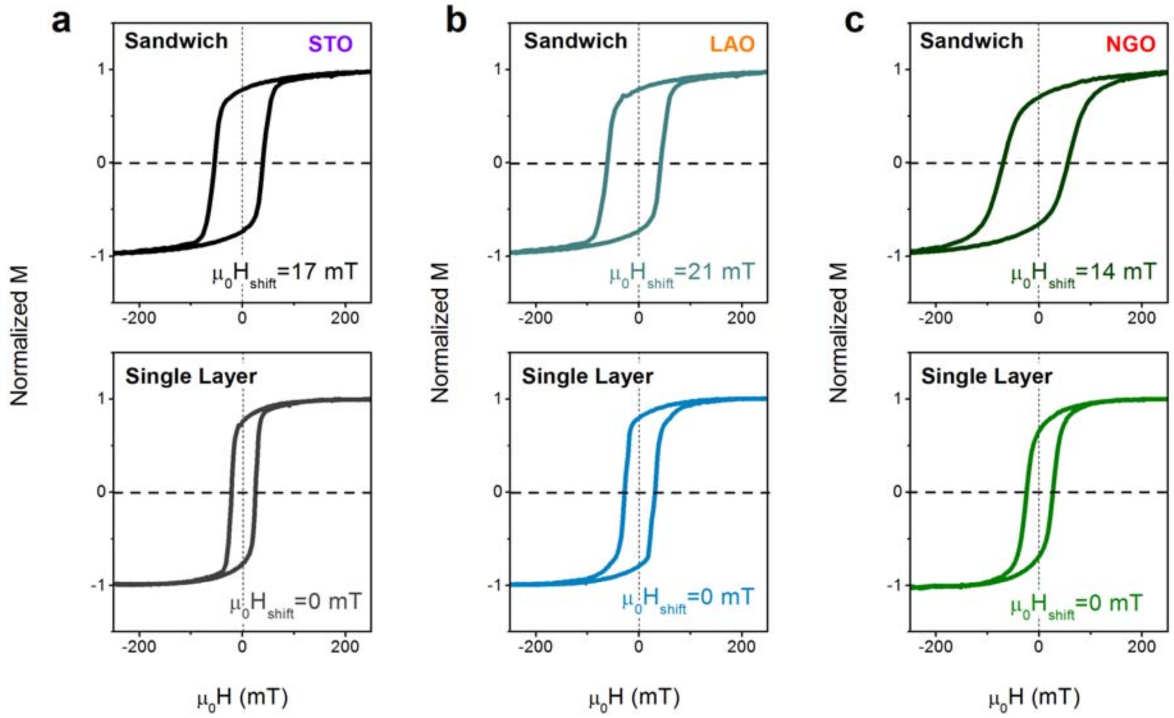

Supplementary Figure 9: M-H loops of (001) LSMO/LAO(4 uc)/LSMO stacks grown on different substrates STO, LAO and NGO. The data are measured at 10 K after field cooling in 7 T. The substrates have lattice parameters of 391, 379 and 387 pm respectively, to be compared with 387 pm for LSAT and LSMO.

Supplementary Note 6

Several sets of LSMO/LAO/STO, LSMO/LAO/LSMO and LSMO/STO/LSMO were fabricated in order to test the reproducibility of the results. In Supplementary Figure 10, all blue curves are the ones in the main text, wine and grey curves present other sets of measurements. As can be seen, the *M-H* loop shift and the Kondo minimum in the *R-T* curves are repeatable, indicating that the key features of this magnetic behaviour are solid, although some small variations are found. Meanwhile error bars are added in every related figure are based on this reproducibility test.

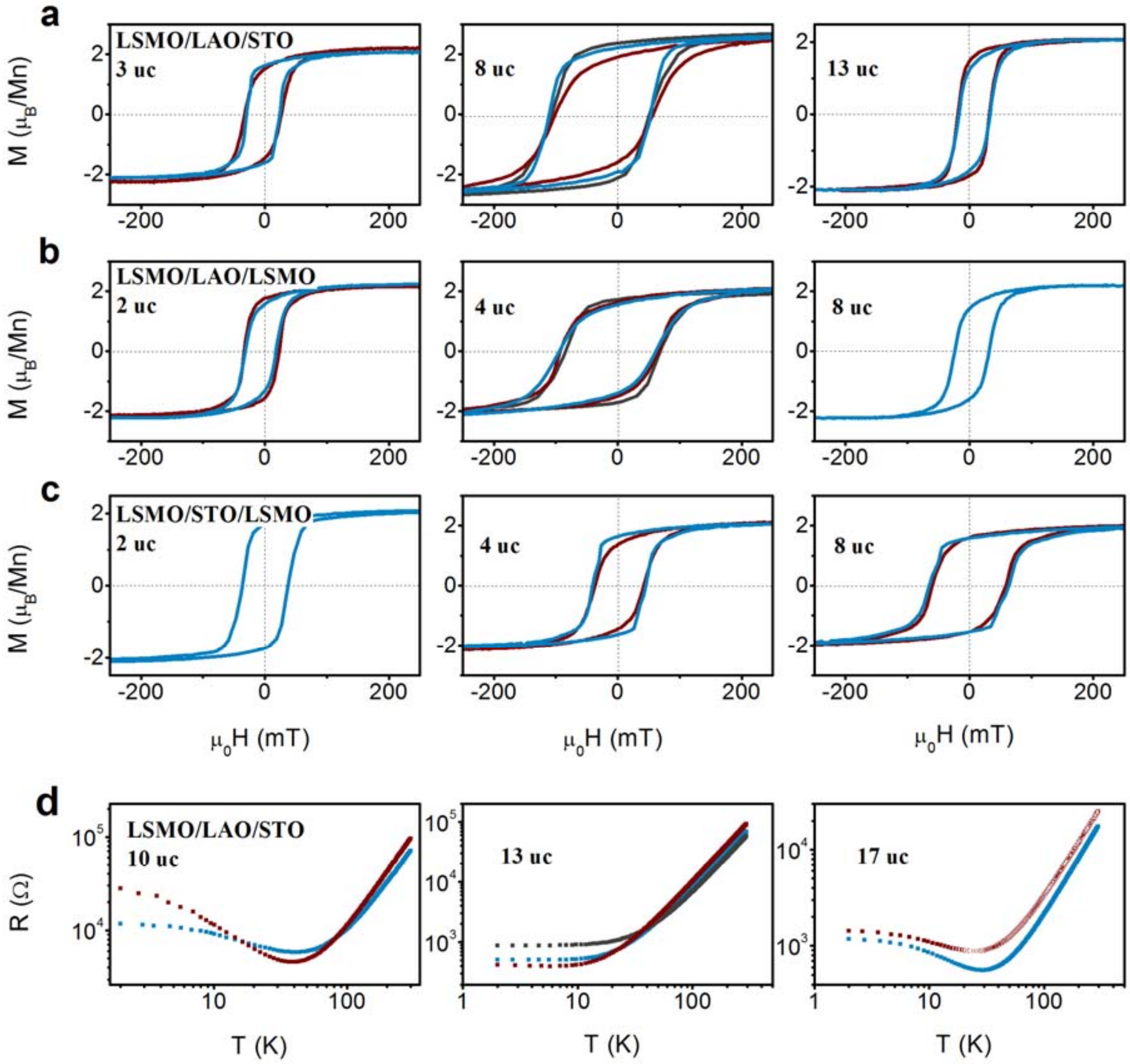

Supplementary Figure 10: a, b and c, The M-H loops of LSMO/LAO/STO, LSMO/LAO/LSMO and LSMO/STO/LSMO. The blue curves are the data presented in the main manuscript, wine and grey ones show the reproducibility of different sets of samples. d, The reproducibility of Kondo effect in the LSMO/LAO/STO heterostructures.

$t \setminus l$ (nm)	50	200	500
2	0.0028	0.0009	0.0004
5	0.0016	0.0008	0.0004
10	0.0007	0.0006	0.0003

Supplementary Table 1: The growth of the oxides on the substrates is quasi-epitaxial (Supplementary Figure 1), so terraces are the only significant source of roughness. Here is a table of the orange-peel coupling in units of mJ m^{-2}. It appears that dipolar interactions and orange-peel coupling of the two LSMO films can definitely be ruled out.

SUPPLEMENTARY REFERENCES

1. Kools, J. C. S., Rijks, T. G. S. M., De Veirman, A. H. M. and Coehoorn, R, On the ferromagnetic interlayer coupling in exchange-biased spin-valve multilayers, *IEEE Trans. Magnetics* **33**, 4513-4521 (1995).
2. Wang, X. *et al*. Magnetoresistance of two-dimensional and three-dimensional electron gas in $LaAlO_3/SrTiO_3$ heterostructures: Influence of magnetic ordering, interface scattering, and dimensionality. *Phys. Rev. B.* **84**, 075312 (2011).
3. Ben Shalom, M. *et al*. Anisotropic magnetotransport at the $SrTiO_3/LaAlO_3$ interface, *Phys. Rev. B.* **80**, 140403(R) (2009).

4. Lee Menyoung, *et al*. Electrolyte gate-controlled Kondo effect in $SrTiO_3$, *Phys. Rev. Lett.* **107**, 256601 (2011).

5. Feng Bi, *et al*. MFM experiments on gated $LaAlO_3/SrTiO_3$ heterostructures. *Nat. Commun.* **5**, 5019 (2014)

6. Kalisky Beena, *et al*. Critical thickness for ferromagnetism in $LaAlO_3/SrTiO_3$ heterostructures, *Nat. Commun.* **3**, 922 (2012).

7. Vaz, C. A. F, Electric field control of magnetism in multiferroic heterostructures, *J. Phys. Condens. Matter.* **24**, 333201 (2012).

8. Vaz, C. A. F., Walker, F. J., Ahn, C. H. and Ismail-Beigi, S. Intrinsic interfacial phenomena in manganite heterostructures, *J. Phys. Condens. Matter.* **27**, 123001 (2015).

9. Aguado-Puente Pablo and Junquera Javier, First-principle study of metal-induced gap states in metal/oxide interfaces and their relation with the complex band structure, *MRS Comm*. **3**, 191 (2013).
